# Soil Microorganisms Alleviate the Allelochemical Effects of a Thyme Monoterpene on the Performance of an Associated Grass Species

**DOI:** 10.1371/journal.pone.0026321

**Published:** 2011-11-17

**Authors:** Bodil K. Ehlers

**Affiliations:** Institute of Biology, University of Southern Denmark, Odense, Denmark; Freie Universität Berlin, Germany

## Abstract

**Background:**

Plant allelochemicals released into the soil can significantly impact the performance of associated plant species thereby affecting their competitive ability. Soil microbes can potentially affect the interaction between plant and plant chemicals by degrading the allelochemicals. However, most often plant-plant chemical interactions are studied using filter paper bioassays examining the pair-wise interaction between a plant and a plant chemical, not taking into account the potential role of soil microorganisms.

**Methodology/Principal findings:**

To explore if the allelopathic effects on a grass by the common thyme monoterpene “carvacrol” are affected by soil microorganisms. Seedlings of the grass *Agrostis capillaris* originating from 3 different thyme sites were raised in the greenhouse. Seedlings were grown under four different soil treatments in a 2*2 fully factorial experiment. The monoterpene carvacrol was either added to standard greenhouse soil or left out, and soil was either sterilized (no soil microorganisms) or not (soil microorganisms present in soil). The presence of carvacrol in the soil strongly increased mortality of *Agrostis* plants, and this increase was highest on sterile soil. Plant biomass was reduced on soil amended with carvacrol, but only when the soil was also sterilized. Plants originating from sites where thyme produces essential oils containing mostly carvacrol had higher survival on soil treated with that monoterpene than plants originating from a site where thyme produced different types of terpenes, suggesting an adaptive response to the locally occurring terpene.

**Conclusions/Significance:**

The study shows that presence of soil microorganisms can alleviate the negative effect of a common thyme monoterpene on the performance of an associated plant species, emphasizing the role of soil microbes in modulating plant-plant chemical interactions.

## Introduction

Allelochemicals released into the soil by plants can affect the performance of interacting plant species [1]. Allelochemicals can act as selective agents driving adaptation in associated plants species to cope with the compounds released by their “chemical neighbor” [2], [3], [4]. In this context, the soil microbial community can potentially affect the interaction between plants and plant chemicals as soil microorganisms may degrade allelochemicals after entering the soil [5]. Most studies examining the effect of plant allelochemicals use bioassays where the effect of a chemical on seed germination and growth is tested in isolated petri dishes. By doing so these studies do not examine the effects of soil microorganisms [5].

Terpenes are the most common group of plant secondary compounds and are produced by a vast amount of plants – both herbs and trees [6], [7]. These compounds can have both inhibitory and stimulating effect on a number of associated organisms including plants, herbivores and microorganisms [7].

Many aromatic plants of the family *Lamiacae* produce monoterpenes as a main constituent of their essential oils. These monoterpenes are known for their antimicrobial activity [8], [9], [10] mainly through their growth inhibitor effect on bacteria and fungi. However, it has been shown that some bacteria can decompose terpenes and use some of them as a carbon source [8], [9], [10]. One genus well known for its production of monoterpenes is *Thymus*. In some thyme species distinct chemotypes can be identified, where an individual plant often produces a single specific dominant monoterpene making up 60–80% of the essential oils that confer thyme plants their characteristic smell [11]. Thyme monoterpenes enter the soil via leaf leachates, and are known to affect the performance of associated plants [12], [13]. In general, the monoterpenes reduce seed germination and plant growth, but it has been shown that associated plants can adapt to their specific local thyme monoterpene [3], [14]. Studies on the impact of thyme monoterpenes on plant growth have been performed using non-sterile soil, either standard greenhouse soil where monoterpenes were manually added, or soil collected from natural sites. The documented effect of the thyme monoterpenes on plant performance therefore included the interaction with the soil microbial community.

The main purpose of this study was to examine if the effect of a single common thyme monoterpene - carvacrol - on the performance of an associated grass, was affected by the presence of soil microorganisms. In Denmark, the grass *Agrostis capillaris* is commonly found in dry grasslands where it co-occurs with *T. pulegioides*. *Thymus pulegioides* produces the monoterpene “carvacrol” as main constituent of its essential oil, and this monoterpene can make up between 50–80% of the total constituent of the oil [15]. *Agrostis capillaris* is also found co-occurring with another thyme species, *T. serpyllum*. The essential oil of *T. serpyllum* is not as pure as in *e.g. T. pulegioides*, and usually consists of a mix of 2–3 different terpenes where carvacrol is not a dominant component ([11], Keefoverring, Grøndahl & Ehlers *unpubl. data*).

## Materials and Methods

### Experimental design

Plants originating from different maternal seed families of *Agrostis capillaris* were grown in individuals pots in a combination of two different types of soil treatments: Soil that was either treated with the thyme monoterpene “carvacrol” or not, and on soil that was either sterilized or not. Plants were grown in a fully factorial design in the greenhouse.

### Plant material

*Agrostis* seeds were collected in August 2008 from different maternal plants growing at three natural sites where they co-occurred with thyme plants, (often growing in the middle of thyme tuffs). At two sites (hereafter named TP1 and TP2) *Agrostis* grows together with *Thymus pulegiodes* producing the monoterpene carvacrol as the main constituent of its oil [15]. At the third site (TS1), *Agrostis* grows with *T. serpyllum*, which produces oil that is a mix of different terpenes, dominated by sesquiterpens and β-caryophyllene (Keefoverring, Grøndahl & Ehlers *unpubl. data*). Collected seeds were stored in paper bags in a dry room until their use the following spring.

Using maternal seed families rather than bulk samples of seeds allow testing for differences in performance among seed families within populations. Albeit confounded with maternal effects [16], detection of significant differences among families suggests presence of genetic variation for performance.

### Soil preparation

Standard greenhouse soil (Pindstrup no 1) was steam sterilized and kept in sealed plastic bags until use. A standard fertilizer mix (N-P-K) was subsequently added to both sterilized and non-sterilized soil, corresponding to 0.078 g N, 0.039 g P, and 0.13 g K per litre soil.

Soil containing the thyme monoterpene carvacrol was prepared by adding 40 µl of liquid pure carvacrol (Sigma Aldricht)/100 g soil (dry weight). This concentration of carvacrol (approximately 400 µg g^−1^ soil) is within the high range of monoterpene concentrations that may be found under natural field conditions [14], [17]. The monoterpene was added to the soil in the following way: Liquid carvacrol was mixed in petri dishes with filter paper (Filtrak paper sheet, 17.95 g m^2^) cut in pieces of approximately 1 cm^2^ and sealed with plastic film for 24 h after which all liquid had soaked into the filter papers. Filter paper was then mixed thoroughly into soil using one single container of soil per treatment. Soil with filter paper was sealed with plastic and left for another 24 h to homogenize the concentration of carvacrol before adding soil to individual pots.

### Germination and transplantation

*Agrostis* seeds were sown in germination trays containing standard greenhouse soil (Pindstrup no. 1). Two weeks after germination, seedlings were transplanted to individual pots (10 cm in diameter). Four seedlings from each maternal family were transplanted to each of the four combinations of soil treatment (soil with and without monoterpene on sterile and non-sterile soil), - each plant grown alone in individual pots, yielding 16 individual plants per maternal family. Care was taken to choose seedlings of similar size in all treatment combinations.

There was variation in the number of maternal seed families from which 16 equal sized seedlings could be obtained. The number of maternal families from each population was 18 (sites TP1 and TP2) and 10 (site TS1) yielding a total of 736 seedlings in individual pots (184 for each of the four soil treatment combinations).

Plants were grown in an unheated glasshouse without addition of plant growth light, so the light range followed the natural photoperiod of the growing season from spring to late summer.

Pots were randomized twice a week to avoid any position effects, and all surviving adult plants were harvested three months after transplant at the end of August 2009. Due to damage of roots when removing soil, root biomass was not included in the analysis.

Dry weight of aboveground biomass of each plant was assessed using a milligram precision balance.

### Data analysis

Survival of plants was analyzed using a logistic regression to test for the effects of monoterpene, soil microorganisms, populations, maternal family (nested within populations), and their interactions. The analysis was performed assuming a binomial distribution of survival data as implemented in the *glm* function of the statistical software *R* (version 2.10.1, R Development Core Team, 2008). Tests of hypothesis regarding the effects of soil treatments, population and their interaction were performed using likelihood ratio tests (LRTs) between nested models and assuming that LRTs statistics where Chi-square distributed.

ANOVA was used to examine for the effect of monoterpene, soil microorganisms, population, maternal family (nested within population), and their interactions on the biomass of plants. The analysis of variance was performed using the software package JMP version 8.0 [18]. This package uses the REML method for variance component and parameter estimation. This method handles naturally unbalanced design and does not rely on approximation for F-test.

## Results

### Survival

Seedling mortality occurred within the first three weeks after transplantation.

**Figure 1:**
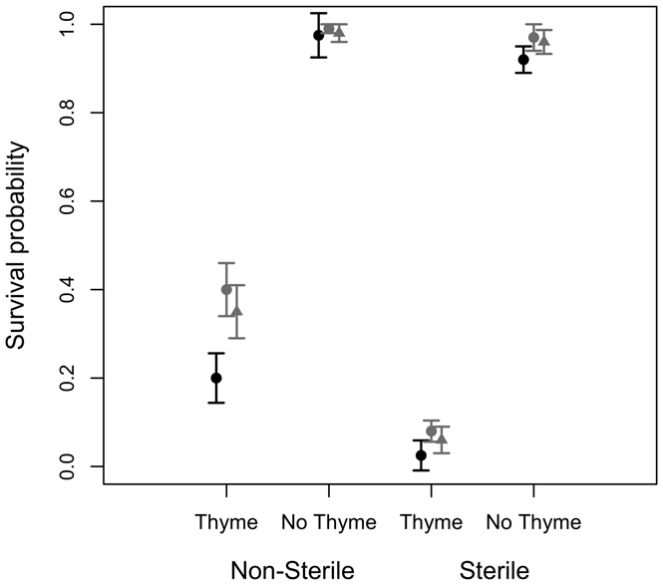
Survival of *Agrostis capillaris* plants. Observed probability (+/− SE) of survival of plants growing in pots where the thyme monoterpene carvacrol is either added to the soil (T) or not added (No T) and where the soil is either sterilized or not. Grey dots: *Agrostis* originating from populations where it co-occurs with *Thymus pulegiodes* (TP), black dots: *Agrostis* originating from a single population where is co-occurs with *T. serpyllum* (TS).

Presence of the thyme monoterpene in the soil greatly reduced the survival of *Agrostis* plants and this effect was reinforced in the absence of soil microorganisms (Fig. 1, Table 1 - interaction between terpene and soil microorganisms). Survival ranged from over 90% in soil without terpene (both sterile and non-sterile soil) to between 20–40% on non-sterile soil treated with monoterpene to as low as 3–8% on sterile soil treated with monoterpene (Fig. 1).

**Table 1:** Summary of the logistic regression analysis on survival of *Agrostis* plants.

Source	df	Deviance	df Residual	Deviance (P>(Chi))
Monoterpene	1	526.34	182	237.04***
Soil	1	45.74	181	191.30 ***
Population	2	10.50	179	180.81 **
Fam(Population)	43	76.17	136	104.64**
Monoterpene*Soil	1	4.41	135	100.23*
Soil*Population	1	19.06	133	81.17***
Monoterpene*Population	1	4.60	131	76.57
NULL	183	763.38		

Significance of test deviance is indicated by:

*P<0.05;

**P<0.01;

***P<0.001.

A significant population effect showed that survival differed among plants originating from different sites. *Agrostis* plants from the *T. serpyllum* site had generally a lower survival rate compared to plants from either of the two *T. pulegioides* sites, but this difference was only present on soil treated with the *T. pulegioides* monoterpene (Fig. 1, compare black triangles with grey symbols on T-soil vs No T soil).

The analysis also revealed a significant variation among maternal seed families (nested within populations) for survival rates. The mortality on sterile soil amended with monoterpene was so high that very little power was left to test if variation among maternal families in survival also varied with presence/absence of soil microbes.

### Biomass

**Figure 2:**
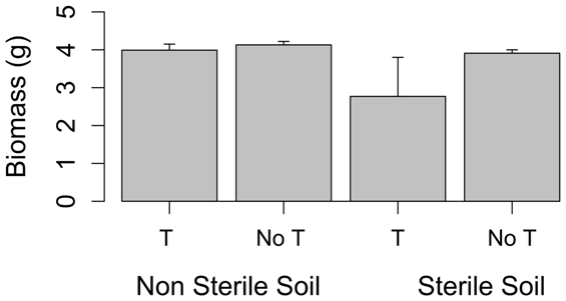
Biomass of *Agrostis capillaris* plants. Biomass (+/− SE) of plants growing in pots with the thyme monterpene carvacrol added to the soil (T) or not (no T) and on soil which was either sterilized or not.

The ANOVA revealed a significant effect of both terpene, soil microorganisms and their interaction on the biomass of *Agrostis*. The addition of monoterpene to the soil reduced biomass of plants but only on sterile soil (Table 2; Fig. 2). The effect of maternal family indicates a variation in biomass among the different seed families within populations. As for survival, the high mortality of plants on sterile soil containing monoterpene excluded the possibility to test if differences in biomass among maternal families varied among sterile and non-sterile soil.

**Table 2:** Summary of ANOVA on plant biomass.

Source	DF	Sum of Squares	F
Monoterpene	1	14.97	14.78***
Soil	1	9.60	9.49**
Population	2	0.68	0.34
Family (Population)	43	100.06	2.30***
Monoterpene*Soil	1	4.55	4.49*
Monoterpene*Population	2	0.58	0.29
Soil*Pop	2	0.13	0.06
Monoterpene*Soil*Population	2	0.46	0.23
Residual	373	377.6	

Significance of F ratio is indicated by:

*P<0.05;

**P<0.01;

***P<0.001.

## Discussion

The main finding of this study is that the soil microorganisms present in the soil can significantly affect the outcome of the plant-plant chemical interaction between a grass and the monoterpene produced by its thyme neighbor plant. Presence of the *T. pulegioides* monoterpene carvacrol in the soil strongly reduced survival of *Agrostis* plants, and mortality was highest when soil microorganisms were not present. In fact, only between 3% (site TS) and 8% (site TP2) of the plants survived the monoterpene treatment when the soil was sterile, compared to survival rates ranging between 20 and 40% when the soil was not sterilized. Survival was uniformly high (>90%) on sterile and non-sterile soil without monoterpene (Fig. 1).

Presence of carvacrol in the soil also reduced biomass of plants, but only when plants grew on sterile soil. There was no difference in biomass of plants growing on soil with and without addition of monoterpene when the soil was not sterilized (Fig. 2). Taken together, these results suggest an alleviating effect of presence of soil microorganisms on the allelopathic effect of a common thyme monoterpene.

The experiment was performed on standard green house soil and identification of soil microorganisms was not the scope of this study. Moreover, it is not likely that the sterilized soil stayed sterile during the entire period of the experiment. Contamination from air and potting may have occurred. However, mortality of seedlings happened during the first weeks after transplantation at a time where the scope for contamination of the sterile soil was minimum.

Overall, survival in this study was much lower compared to previous studies examining the effect of thyme monoterpenes on associated plant species [14], [19]. Albeit using similar monoterpene concentration in the soil treatment, germination of seedlings was in previous studies done on soil also amended with monoterpene. In this study, seeds were germinated on standard green house soil without monoterpene, and seedlings were subsequently transplanted to pots individual of different soil treatments. The transplantation from seedlings, germinating on soil without monoterpene, onto soil containing monoterpene could account for the higher seedling mortality in the present study. Here, it is worth noting that transplantation alone cannot account for mortality as the survival of seedlings transplanted onto soil without the monoterpene did not differ between sterile and non-sterile soil. This also suggests that the germination process is an important screening for tolerance to presence of monoterpene in the soil.

The recovery of carvacrol in the soil under the different treatments was not examined in this study. In natural soil under pine trees, it was found that monoterpene concentration was between 20 and 130 times less than the concentration in fresh pine needles [20]. Assuming a similar dilution factor for thyme leafs, the concentration of carvacrol in soil under carvacrol producing thyme plants may vary between 230–1500 ug per gram soil (see [14] for details). That concentration is within the range used in the experiment reported here. Monoterpenes in soils can be found both in an aqueous phase and as gas in the soil micro air [17], [21]. According to White [17] detection of unbound monoterpenes in soil is difficult and render measures of concentration in natural soil very difficult and often underestimated. It has been suggested that mineral soil may actually act as a sink for monoterpenes [17]. This suggest that the documented effect of these phytochemicals under experimental conditions are also highly relevant in natural conditions.

Soil microbes can have both negative and beneficial effects on plants as pathogens, mutualists, and drivers of nutrient cycling, and plants can in turn affect the microbial community in the soil under the canopy and around the roots [22], [23]. For instance, negative feedbacks of soil microbes have been shown in soil from the home range of plant species where soil microbes have a stronger inhibiting effect on plant growth than soil microbes from outside the home range of plants [24]. Positive feedback typically involves the mutualistic interaction between mychorrhiza or nitrogen fixing bacteria and plants. The pairwise interaction between a plant species and a plant chemical released by a neighbouring plant can indirectly be affected by the action of the soil microbes on the plant chemical. For instance, soil microbes can convert plant allelochemicals to compounds that cause greater inhibition of plant growth than the original compound (see e.g. [25]). Positive indirect effects, on the other hand, can occur when soil microbes obviate the negative effects on plant growth of released plant allelochemicals. This was recently emphasized by Kaur et al. [5], showing that the allelopathic effect of the plant chemical tyrosin on sterile soil was diminished on non-sterile soil. As in the present study, this shows how the bulk soil microbial community can affect the outcome of allelopathic interactions. Indeed, the low concentration in soil of very phototoxic chemical such as catechin and 8-hydroxyquinoline from the roots of *Centaurea maculosa* and *C. diffusa*, respectively has been suggested to be due to the activity of soil microbes [5], [26], [27].

The survival results suggest an adaptive response to the local thyme monoterpene. *Agrostis* plants originating from *T. pulegioides* sites, where they naturally co-exist with thyme producing carvacrol (TP1 and TP2), survived better on soil amended with carvacrol compared to plants originating from a site where thyme produce different types of terpenes (TS1) (Fig. 1).

However as only three populations were tested, only one of which was naïve to carvacrol, the finding of an adaptive response to the home-terpene (carvacrol) can only be seen as suggestive.

In conclusion, the main finding of this study is that bulk soil microbes can alleviate the negative impact of the monoterpene carvacrol on the performance of an associated plant. This finding adds to other recent studies in emphasizing the role of soil microbes for the outcome of plant-plant interactions involving the release of plant allelochemicals to the soil.

## References

[1] Inderjit (1996). Plant phenolics in allelopathy.. The Botanical Review.

[2] Callaway RM, Aschehoug ET (2000). Invasive plants versus their new and old neighbors: a mechanism for exotic invasion.. Science.

[3] Ehlers BK, Thompson J (2004). Do co-occurring plant species adapt to one another? The response of Bromus erectus to the presence of different Thymus vulgaris chemotypes.. Oecologia.

[4] Vivanco JM, Bais HP, Stermitz FR, Thelen GC, Callaway RM (2004). Biogeographical variation in community response to root allelochemistry: novel weapons and exotic invasion.. Ecology Letters.

[5] Kaur H, Kaur R, Kaur S, Baldwin IT, Inderjit (2009). Taking Ecological Function Seriously: Soil Microbial Communities Can Obviate Allelopathic Effects of Released Metabolites.. PLoS ONE.

[6] Gershenzon J, Dudareva N (2007). The function of terpene natural products in the natural world.. Nat Chem Biol.

[7] Langenheim JH (1994). Higher plant terpenoids: A phytocentric overview of their ecological roles.. J Chem Ecol.

[8] Kalemba D, Kunicka A (2003). Antibacterial and Antifungal Properties of Essential Oils.. Current Medicinal Chemistry.

[9] Vokou D, Chalkos D, Karamanlidou G, Yiangou M (2002). Activation of Soil Respiration and Shift of the Microbial Population Balance in Soil as a Response to *Lavandula stoecha* Essential Oil.. Journal of chemical ecology.

[10] Vokou D, Margaris NS (1988). Decomposition of terpenes by soil microorganisms.. Pedobiologia.

[11] Stahl-Biskup E, Stahl-Biskup E, Saez F (2002). Essential oil chemistry of the genus Thymus – a global view..

[12] Tarayre M, Thompson JD, Escarré J, Linhart YB (1995). Intra-specific variation in the inhibitory effects of *Thymus vulgaris* (Labiatae) monoterpenes on seed germination.. Oecologia.

[13] Vokou D, Douvli P, Blionis GJ, Halley JM (2003). Effects of Monoterpenoids, Acting Alone or in Pairs, on Seed Germination and Subsequent Seedling Growth.. Journal of chemical ecology.

[14] Grøndahl E, Ehlers BK (2008). Local adaptation to biotic effects: Reciprocal transplants of species associated with aromatic Thymus pulegioides and T. serpyllum.. J Ecol.

[15] Grøndahl E, Keefover-Ring K, Ehlers BK (2008). New 4-thuyanol chemotype detected in large thyme (Thymus Pulegioides L.) growing wild in Denmark.. J Ess Oil Res.

[16] Lynch M, Walsh B (1998). Genetics and analysis of quantitative traits.

[17] White CS (1991). The role of monoterpenes in soil nitrogen cycling processes in ponderosa pine.. Biogeochem.

[18] SAS Institute I (2008). JMP Users Guide.

[19] Jensen C, Ehlers B (2010). Genetic variation for sensitivity to a thyme monoterpene in associated plant species.. Oecologia.

[20] Wilt FM, Miller GC, Everett RL, Hackett M (1993). Monoterpene concentrations in fresh, senescent, and decaying foliage of singleleaf pinyon (*Pinus monophylla* Torr. & Frem.: Pinaceae) from the western Great Basin.. Journal of Chemical Ecology.

[21] Pavolainen L, Kitunen V, Smolander A (1998). Inhibition of nitrification in forest soil by monoterpenes.. Plant Soil.

[22] Bever JD (2003). Soil community feedback and the coexistence of competitors: conceptual frameworks and empirical tests.. The New phytologist.

[23] Van der Putten WH, Vet LEM, Harvey JA, Wöckers FL (2001). Linking above- and belowground multitrophic interactions of plants, herbivores, pathogens, and their antagonists.. Trends in ecology & evolution (Personal edition).

[24] Callaway RM, Thelen GC, Rodriguez A, Holben WE (2004). Soil biota and exotic plant invasion.. Nature.

[25] Inderjit, Lambers H, Colmer T (2005). Soil microorganisms: An important determinant of allelopathic activity..

[26] Inderjit, Pollock JL, Callaway RM, Holben W (2008). Phytotoxic Effects of (6)-Catechin In vitro, in Soil, and in the Field.. PLoS ONE.

[27] Perry L, Thelen G, Ridenour W, Callaway R, Paschke M (2007). Concentrations of the Allelochemical (±)-Catechin In *Centaurea maculosa*; Soils.. Journal of chemical ecology.

